# Phospholipase A2-Responsive Phosphate Micelle-Loaded UCNPs for Bioimaging of Prostate Cancer Cells

**DOI:** 10.1038/s41598-017-16136-4

**Published:** 2017-11-22

**Authors:** Mirkomil Sharipov, Salah M. Tawfik, Zayakhuu Gerelkhuu, Bui The Huy, Yong-Ill Lee

**Affiliations:** Department of Chemistry, Changwon National University, Changwon, 641-773 Korea

## Abstract

We report the effective synthesis of biocompatible upconversion nanoparticles (UCNP)-loaded phosphate micelles and successful delivery of UCNPs to prostate cancer cells via secreted phospholipase A2 (sPLA-2) enzyme cleavage of the loaded micelles for the first time. The activity of the (sPLA-2) enzyme toward the synthesized micelles was investigated and confirmed by LC-MS. TEM results showed that the micelles have a size distribution of 80 to 150 nm, whereas UCNP-loaded micelles range from 200 to 350 nm, indicating the successful loading of UCNPs. The selective release of UCNPs to prostate cancer cells rather than other cells, specifically cervical cancer cells, was observed and confirmed by a range of bioimaging studies. Moreover, cytotoxicity assays confirmed the biocompatibility of the UCNP-loaded micelles.

## Introduction

The diagnosis of cancer, especially in its early stage, has received a tremendous amount of attention due to the emergence of more than 14 million new cancer cases worldwide each year^1^. Imaging methods using upconversion nanoparticles (UCNPs) have been proposed as a prospective diagnosis tool due to their ability to absorb near-infrared (NIR) radiation and transform it into visible light by an upconversion process of multiple-photon absorption^2–4^. This characteristic feature of UCNPs make it an excellent candidate for bioimaging applications because biological tissue has an optical window situated in the range of 650 to 1350 nm, and NIR wavelengths are able to penetrate more efficiently into biological tissues while exhibiting low cellular photodamage^5,6^. Moreover, these nanoparticles are less prone to photobleaching and have low self-quenching properties^7^. However, employing these nanoparticles in biological systems remains a significant challenge due to their high toxicity and low dispersion in biological media. To address these concerns, the surface modification of nanoparticles generally involves the use of biocompatible chemicals and biological entities such as aptamers, antibodies, sugars, and folic acid to reduce cytotoxicity towards non-cancerous cells by increasing their selectivity toward specific tumor cells and improving their dispersion^8–14^.

Although the biocompatibility of these nanoparticles has been improved upon, designing an effective and highly selective delivery system using UCNPs remains a challenge due to complicated methods of distribution, metabolism, and selective delivery at the target site. Various significant efforts have been devoted to developing delivery systems with a controlled release using exogenous or endogenous stimulations^15–17^. Some previous reports have taken advantage of the acidic pH of cancer cells due to their high proliferation activity. This abnormal acidity in the microenvironment of tumors triggers the release mechanism of several nanocarriers. For example, an immediate dissociation of micelles formed from PEG–poly(β-amino ester) under acidic pH conditions (pH 6.4–6.8) resulted in the liberation of the anticancer drug camptothecin^18^. Other reported methods of drug delivery utilize the altered expression of specific enzymes that directly relate to disease development^19–21^. In these reports, enzyme-specific cleavage of the delivery system results in the release the drugs to target cites that exhibit an over-expression of the specific enzyme. This triggering method has become increasingly popular in the field of stimuli-responsive drug delivery systems due to the potential for high selectivity of drug release into targeted diseased cells.

The diagnostic and screening methods for prostate cancer are limited and controversial. Prostate-specific antigen (PSA) is a widely used biomarker for early stages of prostate cancer, and several sensing methods for PSA have been reported^22,23^. Although an elevated level of PSA corresponds to abnormalities in prostate tissue, solely monitoring PSA can also result in over-diagnosis because most prostate cancer is asymptomatic. Therefore, a low percent of patients with elevated levels of PSA are diagnosed with prostate cancer. It has recently been reported that an over-expression of secreted phospholipase A2 (sPLA-2) in biological systems contributes to the proliferation of prostate cancer cells. Carter *et al*. reported an abnormal sPLA-2 over-expression of 20-fold in primary, high-grade prostate cancer cells^24,25^. sPLA-2 is an enzyme that catalyzes the hydrolysis of phospholipids at the sn-2 position, producing fatty acids and lysophospholipids^26^. Therefore, sPLA-2 could play a significant role in an efficient and highly selective drug delivery system due to its enzymatic activity for the digestion of phospholipids and over-expression in prostate cancer cells.

**Figure 1:**
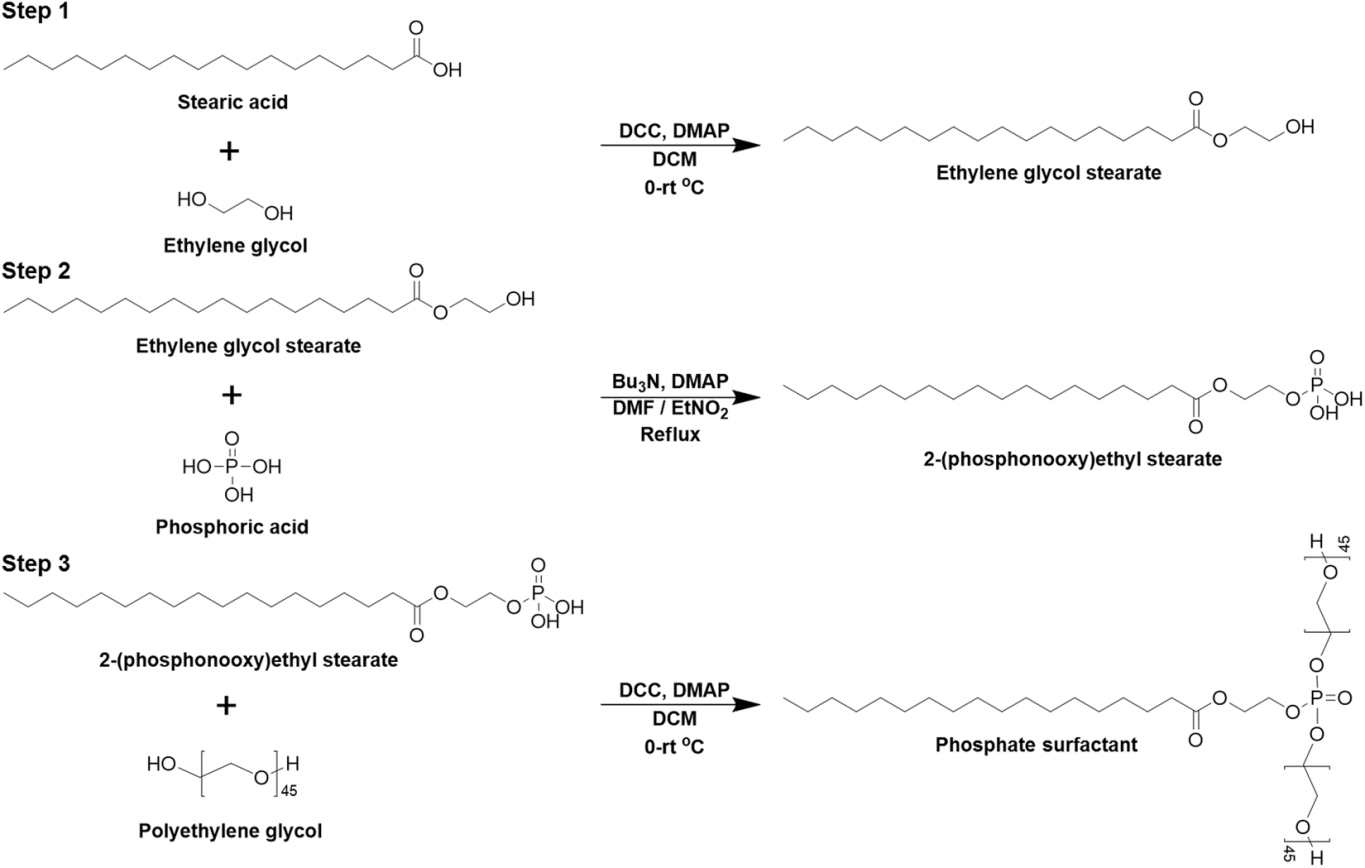
Synthesis of phosphate surfactant. (Step1) Steglich esterification of stearic acid with ethylene glycol. (Step 2) Monophosphorylation of ethylene glycol stearate promoted by the nucleophilic base. (Step 3) PEGylation of phosphate through esterification of Steglich.

Based on the recent discoveries and remaining challenges encountered in the field of bioimaging, we have developed novel UCNP-loaded phosphate micelles which can be cleaved by the secreted phospholipase A2 (sPLA-2) enzyme, allowing the release and delivery of UCNPs directly to prostate cancer cells for the first time. The ability to release UCNPs in a precise location provides several advantages including the efficient delivery of UCNPs in low concentrations, an increase in dispersion, and a remarkably high selectivity to prostate cancer cells. Correspondingly, these benefits will reduce biological side effects that other delivery systems have faced. Nanoparticles were prepared by a hydrothermal method (Supplementary Information, Figure S1), and the delivery system was developed in the form of phosphate micelles. Phosphate surfactant was synthesized from the biocompatible materials stearic acid (SA), ethylene glycol (EG), and phosphoric acid (PA), as shown in Fig. 1.

## Materials and Methods

### Chemicals and Materials

Stearic acid (reagent grade 95%), polyethylene glycol (Mw 2000), dicyclohexylcarbodiimide (DCC, 99%), 4-(Dimethylamino)pyridine (DMAP, 98%), tributylamine (99%), malonic acid (MA, 90%), paraformaldehyde and phospholipase A2 from bee venom (600–2400U/mg) were purchased from Sigma-Aldrich. Lu_2_O_3_ (99.99%), Yb_2_O_3_ (99.99%), Gd_2_O_3_ (99.99%) and Er_2_O_3_(99.99%) were obtained from Minmetals Rare Earth Co. Ltd. Ammonium fluoride (NH_4_F), sodium hydroxide NaCl (reagent grade, ≥98%), HCl (37%), Sodium carbonate (Na_2_CO_3_) were purchased from Alpha Aeser. The distilled water used in all experiments had a resistivity higher than 18 MΩ.cm^−1^ by a Milli-Q water purification system (Millipore Corp., MA, USA). All chemicals and solvents were used as received without further purification. Epithelial prostate carcinoma, 22Rv1 cancer cells, were purchased from Sigma-Aldrich (European Collection of Cell Cultures). KB (HeLa contaminant, carcinoma) cell line was purchased from the Korean cell line bank. The Hela cell line was a kind gift from the Bio-Health department (Changwon National University). DMEM (Dulbecco’s modified Eagle medium) supplemented with L-glutamine, FBS (fetal bovine serum), Pen/Strep (10.000 µg/mL, 10.000 units/mL) and phosphate buffer solution (PBS) was purchased from Thermo Fisher company. The Nunc Lab-Tek Chamber Slide system was purchased from Sigma-Aldrich.

### Characterization

Fourier-transform Infrared (FT-IR) measurements were performed on an FT-IR-6300 spectrophotometer ((Jasco, Japan)) using the ATR crystal technique.^1^H-NMR of the synthesized products was acquired with a Bruker uxNMR 400 MHz instrument. Transmission electron microscopy (TEM) images were recorded on a JEM-2100F transmission electron microscope (JEOL, Tokyo, Japan) operating at 200 kV. TEM samples were prepared by dropping a pre-prepared solution of the micelles and UCNP-loaded micelles in ethanol, onto amorphous carbon-coated copper grids with the excess solvent evaporated. The UC photoluminescence emission spectra were recorded with a fluorescence spectrophotometer (Acton SpectraPro 750-Triplet Grating Monochromator) equipped with a CCD detector (Princeton EEV 1024 × 1024 and PI-Max 133 Controller), and a 980 nm semiconductor CW laser diode (LD) was used as the excitation source. The bio-imaging application was performed on a fluorescence microscope (Olympus BX51) equipped with a CCD detector (PixeLINK PL-A642), and a 980 nm semiconductor CW laser diode was used as the excitation source.

### Synthesis of UCNPs

High quality carboxyl-functionalized NaLuF4:Gd^3+^/Yb^3+^/Er^3+^ upconversion nanoparticles were prepared by the facile one-step hydrothermal method in aqueous solution. In the well-agitated solution of deionized water containing NaCl, malonic acid (MA) and a pre-prepared solution of RECl_3_ was added to a stoichiometric amount of NH_4_F. Then, the solution was transferred to a Teflon-lined autoclave and heated to 200 °C for 8, 12, and 24 h. Obtained nanoparticles were collected by centrifugation, washed with ethanol then with DI water several times and dried at 50 °C for 24 h. After the characterization of UCNPs by XRD, FTIR, FE-SEM and FTIR methods, it was revealed that the 12 h reaction time is optimal, that is to say, the nanoparticles with smaller sizes and higher emission properties. The size of 12 h UCNPs are relatively smaller compared with that of 24 h UCNPs. The intensity of upconversion emission of 12 h UCNPs was relatively higher than that of 8 h UCNPs. Therefore, 12 h reaction time has been chosen as an optimal time to get nanoparticles with upconversion intensity as high as possible, along with keeping the size of nanoparticles as small as possible.

### Synthesis of phosphate surfactant

#### Step 1: Synthesis and purification of ethylene glycol stearate

The synthesis of the ethylene glycol stearate was conducted through Steglich esterification of the carboxylic acid^27^. This method is considered to be a green synthesis approach because of the low reaction temperature (0-rt °C). Stearic acid (SA), 0.005 mol (1.422 g), was dissolved in 30 mL of dichloromethane (DCM), and three equivalents (0.9310 g) of ethylene glycol (EG) was added and stirred in an ice-water bath. To accelerate the reaction, 4-dimethylaminopyridine (DMAP) was used as a catalyst. After the reaction medium was cooled down to 0 °C, the prepared solution containing 2.27 g of dicyclohexylcarbodiimide (DCC) in 20 mL DCM was added dropwise. After DCC was added, the ice-water bath was removed, and the reaction medium was stirred at room temperature for two days. Upon completion, the formation of solids corresponding to dicyclohexylurea (DCU) was observed. To purify the product, the solution was first filtered then washed with a saturated solution of sodium carbonate three times. After each washing, the product was filtered to remove the formed solids and to simplify the separation of the organic and aqueous phases. Afterward, the product was washed twice with a dilute solution of 1 N hydrochloric acid. After completing the washing the organic phase, the recrystallization of the product was performed in an ice-water bath to remove the remaining stearic acid. The product was washed with methanol for 5 minutes to get rid of the remaining DCU in the solution.

#### Step 2: Synthesis and purification of 2-(phosphonooxy)ethyl stearate

For the synthesis of the phosphate monoester, we used the dehydrative condensation of phosphoric acid with ethylene glycol stearate promoted by a nucleophilic base, tributylamine^28^. This method is a direct condensation of an equimolar amount of phosphoric acid and ethylene glycol stearate. Since high temperature reactions are not environmentally friendly, an azeotropic solvent DMF/EtNO_2_ was chosen. Ethylene glycol stearate (EGS), 3 mmol (1.18 g), was dissolved in a DMF/EtNO_2_ and stirred at 35–40 °C to dissolve EGS completely. Subsequently, tributylamine (Bu_3_N), 3 mmol (0.71 mL), and Dimethylaminopyridine (DMAP), 10% mol (0.037 g), were added to reaction medium. Finally, phosphoric acid (PA), 3.04 mmol (3.46 mL), was added and heated under azeotropic reflux conditions. A Dean-Stark apparatus was used to eliminate water and consequently control the reaction time. After the reaction medium was cooled down to room temperature, recrystallization of the liquid phase was performed. The product was washed with ethyl acetate to eliminate unreacted 2-(phosphonooxy)ethyl stearate.

#### Step 3: Synthesis and purification of PEGylated 2-(phosphonooxy)ethyl stearate

PEGylation of the phosphate functional group in the 2-(phosphonooxy)ethyl stearate surfactant was attained through Steglich esterification. 2-(phosphonooxy)-ethyl stearate, 0.1 mol (0.195 g), was dissolved in 30 mL of dichloromethane (DCM), and two equivalents (0.9310 g) of polyethylene glycol (PEG) was added and stirred in an ice-water bath. To accelerate the reaction, 4-Dimethylaminopyridine (DMAP), 10% mol, was used as a catalyst. After the reaction medium was cooled down to 0 °C, the pre-prepared solution containing 0.227 g of dicyclohexylcarbodiimide (DCC) in 20 mL DCM was added dropwise. After the DCC was added, the ice-water bath was removed, and the reaction medium was stirred under nitrogen at room temperature for two days. The reaction medium was then filtered, and the solvent was evaporated with a rotary evaporator. The oily product was washed with ethyl acetate to remove any traces of catalyst.

### Critical micellar concentration

CMC was measured through a dye micellization method using pyrene^29^. 0.400 mg of pyrene was dissolved in 10 mL acetone then the solution was diluted to obtain multiple tubes of 1 mL solution with final concentration (6 × 10^−6^ M). Then acetone was evaporated using vacuum oven. Phosphate surfactant solution with different concentration were prepared separately. To obtain a solution with concentration of 2 mM, 0.172 g (3.934 × 10^−5^ mol) of the phosphate surfactant was dissolved in 20 mL water. This solution was then diluted to obtain 10 solutions of different concentrations. Finally, the 10 mL of the solutions with varying concentrations of phosphate surfactant were added to tubes containing pyrene, and solutions were sonicated for 30 minutes. Photoluminescence was measured for each solution, samples were excited at 335 nm, and the intensity at λ_max_ for I_1_ and I_3_ were recorded. Ratio of (I_3_/I_1_) were calculated. In our case the intensities of emission at 382 (I_1_) and 420 (I_3_) were recorded.

### Specific activity of sPLA-2 on surfactant

The digestion was performed in the presence of Ca^2+^ since the sPLA-2 enzyme depends on the concentration of calcium ions. First, ten aliquots with sPLA-2 concentrations of 1.2 × 10^6^ − 4.8 × 10^6^ U/L were prepared and stored in −20 °C to preserve the initial specific activity of the enzyme. Phosphate surfactant solutions (1.22 mM) were prepared in PBS at pH = 7.2 with calcium chloride (2 mM). A dilute solution of sPLA-2 (60–240 U/L) was added to the phosphate surfactant solution. Solutions with and without the enzyme were kept in a water bath for overnight at 37 °C. To confirm the hydrolysis reaction, the solutions were then extracted with dichloromethane (DCM) and further washed with Milli-Q water three times to eliminate salts. The extracted and dried product was then dissolved in 1:1 DCM/ethanol and analyzed using mass spectrometry.

### Encapsulation of UCNPs in micelles

Encapsulation of UCNPs in micelles was achieved through the simple sonication of UCNPs with phosphate surfactant at room temperature for 30 minutes. Phosphate surfactant, 2.27 μmol (10 mg), was dissolved in 5 mL PBS at pH = 7.2. Afterwards, 3 mg of UCNPs was added to the solution of phosphate surfactant and sonicated for 30 minutes at 37 °C. Moreover, the dispersion of the MA@UCNPs in different solvents with polarity ranging from 0.1–10.2 was investigated. 3 mg of UCNPs was dispersed in 2 mL of solvent and sonicated for 30 minutes. To compare the effect of the surfactant on the dispersion, methanol and water solution with 6 × 10^−4^ M surfactant was prepared, and the UCNPs were added at same time in all solvents (see Table S1).

### Bioimaging application

HeLa (Human cervical cancer, adenocarcinoma) cell lines, KB (HeLa contaminant, carcinoma) cell lines, and 22Rv1 (Human prostate cancer, carcinoma) cell lines were grown in DMEM (Dulbecco’s modified Eagle medium) supplemented with L-glutamine, MEM (Minimum Eagle’s medium), and RPMI 1640 Medium (ATCC modification), respectively. All media were supplemented with 10% FBS (fetal bovine serum) and 5 mL of Pen/Strep (10.000 µg/mL, 10.000 units/mL) at 37 °C and 5% CO_2_^1–3^. Cells (10^4^ cells/100 μL) were seeded on LAB-Tek chamber slides and allowed to adhere for 24 h. Subsequently, after washing with phosphate buffer solution (PBS), the cells were incubated in the corresponding growing media containing 0.3 mg/mL UCNPs or UCNP-loaded micelles at 37 °C for 1 h under 5% CO_2_ and then washed with PBS sufficiently (3 times) to remove excess nanoparticles. Then cells were incubated in 4% freshly-prepared paraformaldehyde in PBS for 15 mins at room temperature. Subsequently, chamber slides were washed 3 times with PBS and dried at room temperature. Cells were excited by a CW infrared laser at 980 nm, and luminescence signals were detected by the microscope.

### Cytotoxicity test: MTT assay

MTT (3-[4,5- dimethylthiazol-2-yl]-2,5-diphenyltetrazolium bromide) assay was used to verify the cell viability of cancer cells. Cells were seeded into a 96-well cell culture plate at 10^4^/L/well and allowed to adhere for 24 h at 37 °C under 5% CO_2_. UCNPs were dispersed in different growing media depending on the type of cells in various concentrations of 0.300, 0.150 and 0.075 mg/mL. Subsequently, these solutions were added to the wells of the treatment group, and the media alone to the negative control group. Cells were incubated for 24 h at 37 °C under 5% CO_2_. Subsequently, 100 µL of MTT (5 mg/mL) was added to each of the treated and negative control wells of the 96-well assay plate and incubated for an additional 4 h at 37 °C under 5% CO_2_. After the addition of 100 µL/well DMSO-Ethanol solution (1:1), the assay plate was kept at room temperature for 15 minutes. A Tecan Infinite M200 monochromator-based multifunction microplate reader was used to measure the OD 570 (Abs value) of each well with background subtraction at 540 nm. At least three independent experiments were performed in each case. And the cells viability was calculated according previously reported work^30^.

## Results

### Synthesis of phosphate surfactant

**Figure 2:**
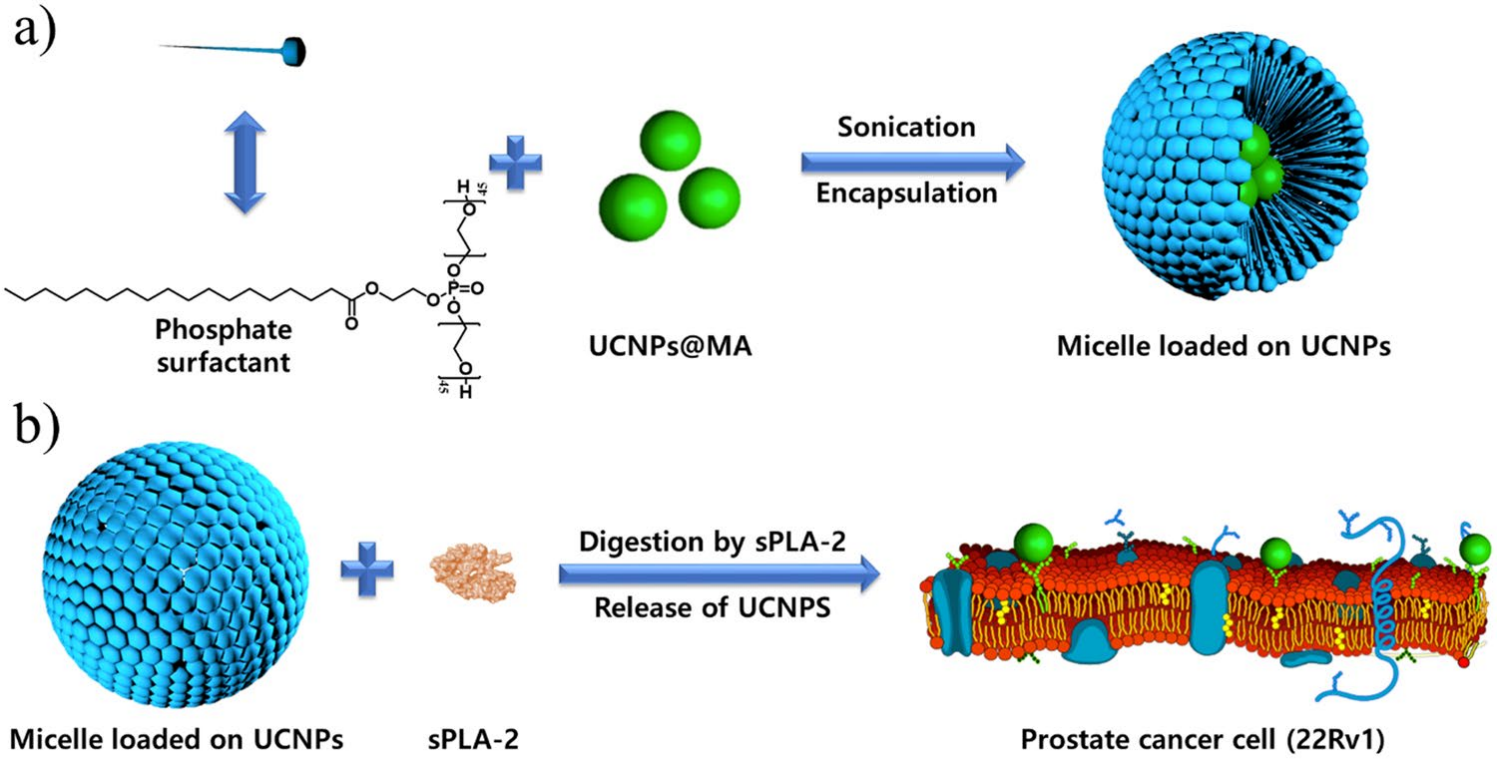
Schematic illustration of encapsulation procedure and release mechanism of UCNPs. (**a**) Encapsulation of UCNPs in novel synthesized phosphate surfactant through sonication at rt. (**b**) Release of UCNPs after a specific cleavage of phosphate surfactant by sPLA-2 enzyme.

We sought to design a new phosphate surfactant to bear a resemblance to phospholipids to facilitate sPLA-2 enzyme recognition and cleavage. This unique characteristic played the main role in the controlled release of UCNPs, as the ester group located between ethylene glycol and the fatty acid was hydrolyzed by the enzyme, and the instant liberation of nanoparticles on the surface of prostate cancer was triggered as illustrated in Fig. 2. The synthesis of the phosphate surfactant was completed in three steps. Bearing in mind that a biological system is composed mainly of water, the hydrophilicity of surfactants was increased by conjugating them to long chains of polyethylene glycol (PEG). Synthesis of the ethylene glycol stearate (EGS) was attained through Steglich esterification of the carboxylic acid (See Figure S3). The purification of the product was performed in methanol for 5 minutes. The molecular structure was confirmed by Fourier transform infrared spectroscopy (FT-IR) and proton NMR (^1^H NMR) (Supplementary Information, Figure S2). In Figure S2, FT-IR spectroscopy reveals a shift in the peak from 1650 cm^−1^ to 1737 cm^−1^, which corresponds to the transformation of the carboxylic acid group into an ester functional group, confirming the successful synthesis of ethylene glycol stearate. Further confirmation of the structure was obtained from the proton NMR (^1^H NMR) of ethylene glycol stearate.

Monophosphorylation of EGS was accompanied by the dehydrative condensation of phosphoric acid and free alcohol group, promoted by tributylamine (Bu_3_N) and DMAP in the azeotropic solvent DMF/EtNO_2_. Figure S2 shows a peak at 1737 cm^−1^ that corresponds to the ester group as well as a peak appearing between 920 cm^−1^ and 1088 cm^−1^ that confirms the presence of P-O-C bonds. Moreover, a peak situated between 1600 cm^−1^ and 1700 cm^−1^ indicates the presence of the O = P-OH group. To confirm the presence of the phosphate group in 2-(phosphonooxy)-ethyl stearate, EDAX was conducted. Elemental analysis showed the presence of the phosphorous atom, and the percentage of the phosphorous atom compared to percentages of carbon and oxygen confirmed the presence of the phosphate group. (Supplementary Information, Figure S4).

PEGylation of the phosphate functional group in 2-(phosphonooxy)-ethyl stearate phosphate was attained in the presence of DMAP and DCC in DCM after being stirred at room temperature for 24 h. FT-IR spectra, Figure S2, confirms the successful PEGylation. The strong peak situated between 3000 cm^−1^ and 2820 cm^−1^ corresponds to the increased number of aliphatic hydrocarbons. The peak at 1737 cm^−1^ is attributed to the ester group formed between the fatty acid and ethylene glycol. Peaks located between 1000 cm^−1^ and 1100 cm^−1^ confirm the presence of P-O-C groups and of polyethylene glycol chains on the phosphorous atom. The peak located between 1600 cm^−1^ and 1700 cm^−1^ is shifted to a lower wavelength with a decrease in intensity due to the coupling of the polyethylene glycol chains with the hydroxyl groups of phosphate. The second step product has ability to partially disperse in water. Therefore, the conjugation of PEG chains was performed to increase the dispersion of micelles in aqueous solution.

### Determination of critical micelle concentration (CMC)

**Figure 3:**
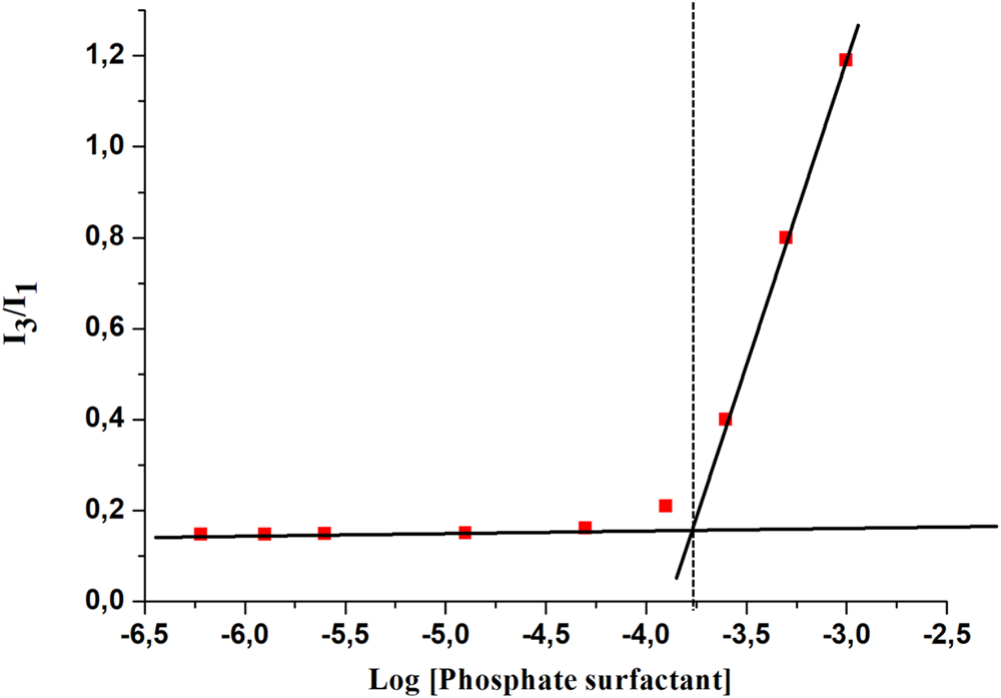
Determination of Critical Micelle Concentration (CMC). The formation of micelles at 1.737 × 10^−4^ M of the surfactant.

To characterize the surfactant properties, CMC was determined through a dye micellization method using fluorescent probe pyrene. Figure 3 shows the plot of intensity ratios (I_3_/I_1_) as a function of the phosphate surfactant concentration. The curves show a gradual rise in the ratios with concentration. The formation of micelles was observed at the concentration of 1.737 × 10^−4^ M of the surfactant. The CMC of our novel surfactant is within the range of other previously reported surfactants^31–33^.

### Activity of sPLA-2 on phosphate surfactant

**Figure 4:**
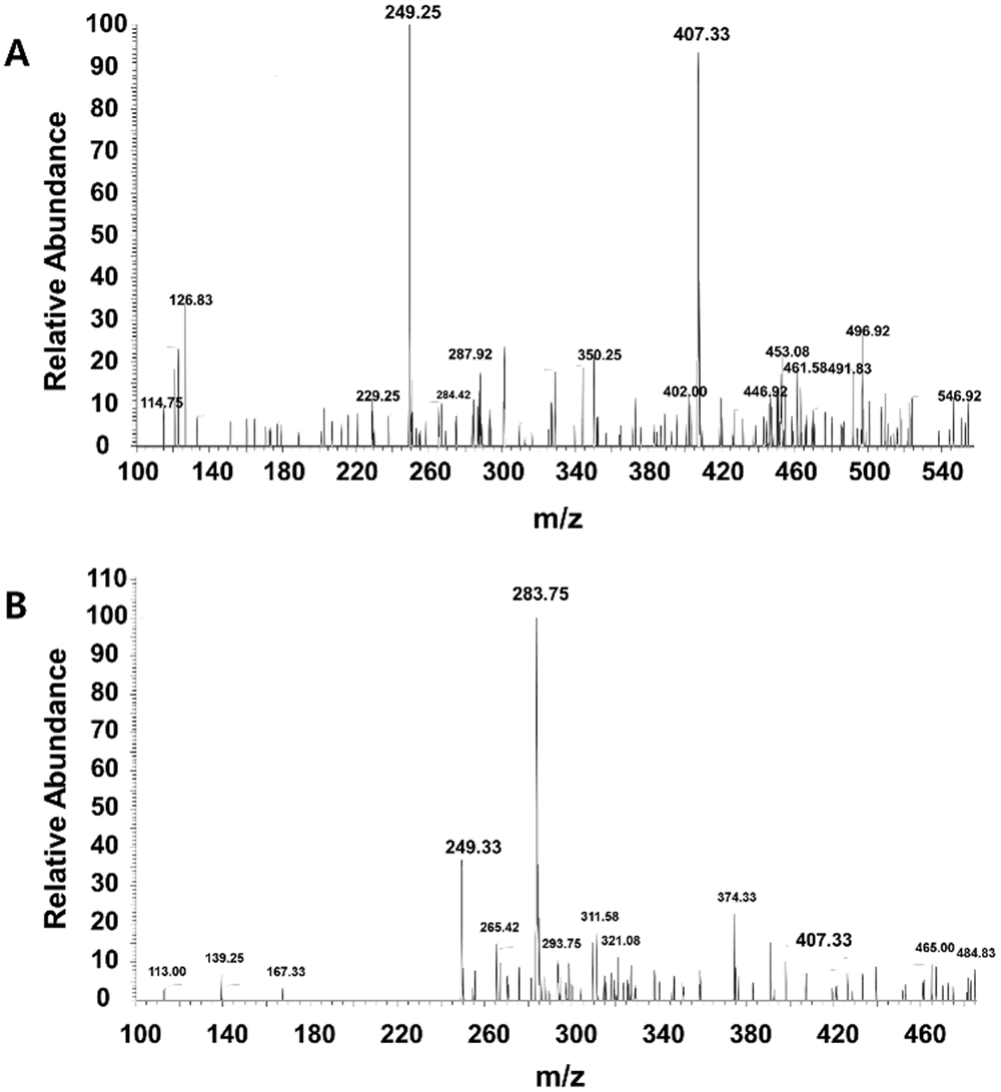
Specific activity of active bee venom sPLA2 enzyme on surfactant. (**A**) Mass spectrum of phosphate surfactant before digestion by active bee venom sPLA-2 enzyme. (**B**) Mass spectrum of phosphate surfactant after digestion by active bee venom sPLA-2 enzyme. Phosphate surfactant at 407.33 m/z. Stearic acid at 283.75 m/z by hydrolysis of the phosphate surfactant.

Since the release of UCNPs is based on the over-expression of the enzyme sPLA-2, an enzymatic digestion was performed to confirm the specific activity of sPLA-2 towards the surfactant. An active bee venom sPLA-2 was used for the digestion of the surfactant and the cleaved fragments were identified using liquid chromatography coupled mass spectrometry (LC/MS). The surfactant, 2-(phosphonooxy)-ethyl stearate was used as a substrate to assess the probability of enzyme digestion. After the addition of the enzyme, the formation of a white suspension was observed and migrated to the surface of the solution. Due to the differences in density, the formed stearic acid migrated to the top of the solution, whereas the non-treated solution didn’t display any physical changes. (Supplementary Information, Figures S5). In Fig. 4, the MS spectrum of a solution containing only the surfactant shows a peak at 407.33 m/z which corresponds to the phosphate surfactant. The signal-to-noise ratio (S/N) of the peak at 407.33 m/z is relatively low, which can be explained by the low concentration of phosphate surfactant due to the extraction step with DCM, where a large amount of phosphate surfactant was likely left in the aqueous phase due to its hydrophilicity. The disappearance of the peak at 407.33 m/z was observed after the injection of the enzyme, indicating the hydrolysis of the phosphate surfactant by the enzyme to form stearic acid which appeared at 283.75 m/z.

### Encapsulation of UCNPs in micelles

**Figure 5:**
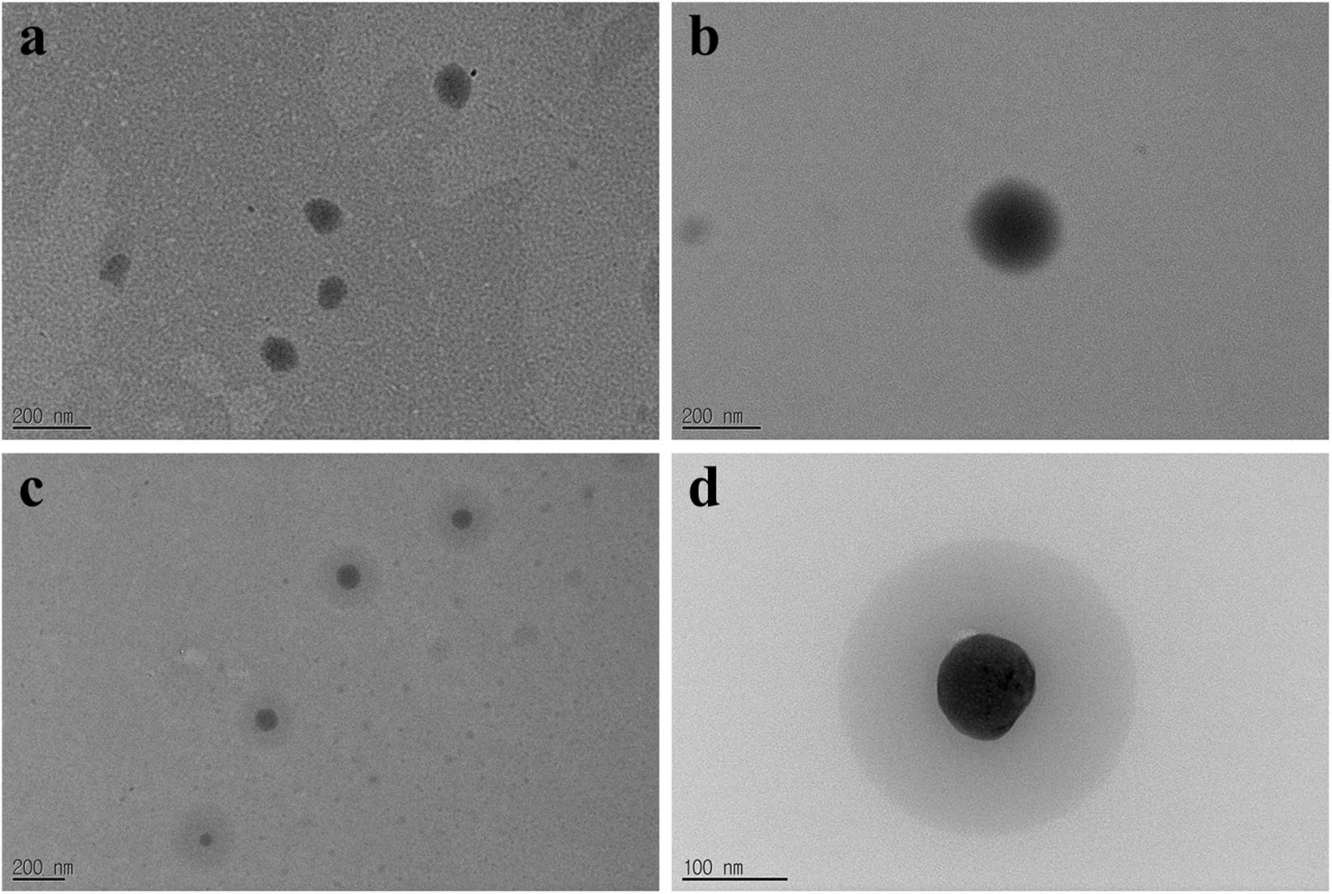
Structural characterization of micelles. TEM images of formed micelles before and after loading of UCNPs. (**a**) Formed micelles without further loading of UCNPs. (**b**) Single micelle without further loading of UCNPs. (**c**) UCNPs-loaded micelles. we can see core shell morphology of UCNPs-loaded micelles. (**d**) Single UCNP-loaded micelle.

The synthesized phosphate surfactant was then used for the encapsulation of nanoparticles through a simple sonication method at room temperature. The UCNP-loaded micelles were characterized through FT-IR and EDAX (Supplementary Information, Figures S6 and S7). In Figure S6, the peak corresponding to the carboxylic functional group of malonic acid appears at 1651 cm^−1^ before and after addition of the surfactant. Consequently, there is no formation of hydrogen bonds between malonic acid and PEG. FTIR spectrum has confirmed the formation of UCNPs-loaded micelles, not by coating with the UCNPs through adsorption. Moreover, the average size of UCNPs through SEM image was found to be 72 nm, see Figure S1. In Fig. 5a,b the TEM image of micelles without UCNPs has showed that the size of micelles is ranged from 80 nm to 150 nm. While, the size of UCNPs-loaded micelles was increased and found to range from 200 nm to 350 nm as shown in Fig. 5c,d. Further evidence is obtained from the TEM image for the encapsulated UCNPs in micelles with core-shell like morphology as in Fig. 5d, indicating the successful loading of UCNPs into the micelles. Figure S8 confirmed that the dispersion of the UCNPs in methanol and water is respectively partial and low, even after long sonication. However, the dispersion of UCNPs is completed within a short time in n-hexane, carbon tetrachloride and chloroform. Moreover, methanol and water solvents containing surfactants showed a complete and fast dispersion of UCNPs in the solution.

### *In vitro* bioimaging application

**Figure 6:**
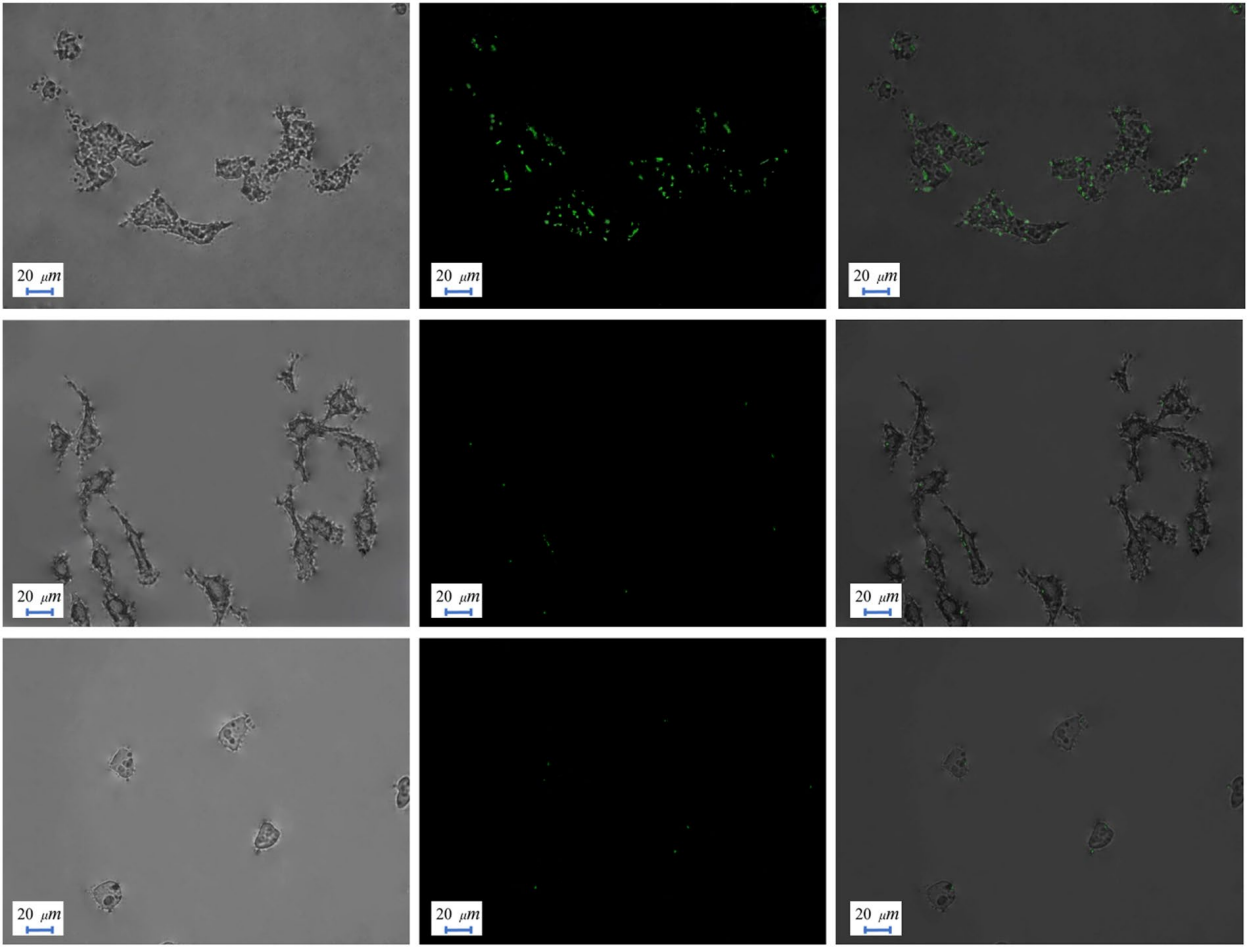
Bioimaging profiles on three different cells. (**a**) 22Rv1 cell line treated with UCNP-loaded micelles. (**b**) HeLa cells treated with UCNP-loaded micelles. (**c**) KB cells treated with UCNP-loaded micelles. High selectivity of nanoparticles towards 22Rv1 cell line is observed by the reaction of over-expressed sPLA-2 enzyme.

To evaluate the selectivity of UCNP-loaded micelles toward sPLA-2-expressing prostate cancer cells, three different cells, HeLa, KB and 22Rv1, were chosen. HeLa (Human cervical cancer, adenocarcinoma) is the most commonly used hard cell line. On the other hand, the KB cell line (HeLa contaminant, carcinoma) is relatively fragile. Both cell lines mentioned above do not over-express the sPLA-2 enzyme, while the 22Rv1 (prostate carcinoma) cell line is known to over-express the sPLA-2 enzyme, and subsequently a high selectivity of UCNPs towards the 22Rv1 cell line was observed. We also confirmed that UCNPs encapsulated with the micelles have a noticeable decrease in toxicity toward all cells through *in vitro* bioimaging assays, especially to sPLA-2 non-expressing cell lines. Since the release of UCNPs is directly related to the sPLA-2 enzyme that is not present in HeLa cells and KB cells, UCNP-loaded micelles have no affinity to these types of cells due to the protective shell from the surfactant. Figure 6 reveals very weak upconversion fluorescence in the HeLa and KB cells. However, the 22Rv1 cell line exhibits an intense upconversion fluorescence, as illustrated in Fig. 6, confirming the delivery of UCNPs to the prostate cancer cell line. Additionally, cells incubated with UCNPs without encapsulation show an intense upconversion fluorescence without selectivity to the 22Rv1 cell line. (Supplementary Information, Figure S9).

### The cytotoxicity assay

The investigation of cytotoxicity is a key factor in determining the potential of employing these materials in practical biomedical applications. To confirm the low cytotoxicity of UCNP-loaded micelles, an MTT assay was conducted on three different cells lines. As expected, after incubating the cells with UCNP-loaded micelles for 24 h, the cell viability was improved after encapsulation of UCNP in micelles. (Supplementary Information, Table S1, and Figure S10) It is important to note there was an observable reduction in the cytotoxicity after the encapsulation of UCNPs with micelles.

## Discussion

Bioimaging application of prostate cancer cells has confirmed the successful synthesis of a novel, water-soluble, biocompatible phosphate surfactant via a simple three-step method that is capable of encapsulating and selectively releasing UCNPs to prostate cancer cells. The structure of the synthesized phosphate surfactant was confirmed through FTIR,^1^H-NMR, and elemental analysis. Critical micellar concentration was determined using a dye micellization method and was found to be 0.1737 mM. Nano sized UCNPs were prepared through a one-step hydrothermal method and characterized through XRD, FE-SEM, FTIR, and photoluminescence techniques. Loading UCNPs onto the micelles was achieved through a simple sonication method at room temperature. UCNPs are distributed throughout the micelles with size ranges between 200 and 350 nm. The enzymatic activity of sPLA-2 towards our developed surfactant was confirmed by digestion with the active bee venom sPLA-2 enzyme and monitored through LC-MS. Finally, bioimaging tests were conducted on three different cell lines to evaluate the selective imaging of prostate cancer cells. HeLa and KB cell lines treated with UCNP-loaded micelles were not wrapped by nanoparticles and, consequently, no upconversion fluorescence was observed. However, 22Rv1 (prostate cancer) cell lines were covered by UCNPs which were liberated from the sPLA-2-cleaved micelles. Furthermore, novel micelles remarkably reduced the cytotoxicity of the nanoparticles, making them a promising prospective imaging agent. The development of an effective delivery system capable of simultaneously transporting imaging agents and anti-cancer drugs will grant a valuable innovation in bioimaging and nanomedicinal applications.

## Electronic supplementary material

Supplementary Information
