# Supplementary material for: Phospholipase A2-Responsive Phosphate Micelle-Loaded UCNPs for Bioimaging of Prostate Cancer Cells
Source: Sci Rep. 2017 Nov 22;7:16073. doi: 10.1038/s41598-017-16136-4 (PMC5700164; doi:10.1038/s41598-017-16136-4)
Supplement: Supplementary file 1 — Supplementary Information [file 41598_2017_16136_MOESM1_ESM.doc]

Supplementary Information

Phospholipase A2-Responsive Phosphate Micelle-Loaded UCNPs for Bioimaging of Prostate Cancer Cells

Mirkomil Sharipov^1^, Salah M Tawfik^1^, Zayakhuu Gerelkhuu^1^, Bui The Huy^1^, Yong-Ill Lee ^*^

^1^Department of Chemistry, Changwon National University, Changwon 641-773, Korea

*Corresponding author (Tel:+82-55-213-3436 Email: [yilee@changwon.ac.kr](mailto:yilee@changwon.ac.kr))

Abstract: Rare-earth upconversion nanoparticles upconversion nanoparticles (UCNP) have been proposed as novel future luminescent labels for bioimaging applications. However, their toxicity, low dispersion, and low selectivity call into question their suitability for nanomedicinal applications. Herein, we report the effective synthesis of biocompatible UCNP-loaded phosphate micelles and successful delivery of UCNPs to prostate cancer cells via secreted phospholipase A2 (sPLA-2) enzyme cleavage of the loaded micelles. The activity of the (sPLA-2) enzyme toward the synthesized micelles was investigated and confirmed by LC-MS. TEM results showed that the micelles have a size distribution of 80 to 150 nm, whereas UCNP-loaded micelles range from 200 to 350 nm, indicating the successful loading of UCNPs. The selective release of UCNPs to prostate cancer cells rather than other cells, specifically cervical cancer cells, was observed and confirmed by a range of bioimaging studies. Moreover, cytotoxicity assays confirmed the biocompatibility of the UCNP-loaded micelles.

Table of Contents

1. Results and discussion
2. Figure S1. Characterization of UCNPs
3. Figure S2. Characterization of the phosphate surfactant. A. FTIR spectra of surfactant for three steps. A) EGS after esterification, b) 2-(phosphonooxy)ethyl stearate after phosphorylation of EGS, and c) Final phosphate surfactant after PEGylation. B) ^1^H NMR of surfactant.
4. Figure S3. Washing step of ethylene glycol stearate (EGS) with methanol
5. Figure S4. Elemental analysis (EDAX) of 2-(phosphonooxy)ethyl stearate
6. Figure S5. Physical changes after enzymatic activity of bee venom sPLA-2 on surfactant
7. Figure S6. FTIR spectra for UCNP-loaded micelles
8. Figure S7. Elemental analysis (EDAX) of UCNP-loaded micelles
9. Figure S8. Dispersion of UCNPs and UCNPs-loaded micelles in different solvents.
10. Figure S9. Bioimaging of Hela and KB Cells with UCNPs without further encapsulation in surfactant micelles
11. Figure S10. Cytotoxicity (MTT assay) of UCNP-loaded micelles toward HeLa cell line, KB cell line, and 22Rv1 cell line

Results and Discussion

Synthesis and characterization of UCNPs

Carboxyl-functionalized NaLuF4:Gd^3+^/Yb^3+^/Er^3+^ upconversion nanoparticles were characterized by XRD, FT-IR, photoluminescence, and FE-SEM. In figure S1, the peaks of the XRD pattern of the NaLuF_4_:Gd^3+^/Yb^3+^/Er^3+^ UCNPs were well indexed to the reference cubic phase NaLuF_4_ pattern (JCPDS-27-0725), and no other impurity peaks were detected. The carboxyl-functionalized surface of NaLuF_4_:Gd^3+^/Yb^3+^/Er^3+^ NPs was identified by FT-IR spectra. The broad absorption band at around 3435 cm^-1^ corresponds to the O-H stretching vibration. A sharp peak centered at 1632 cm^-1^ is attributed to the carboxylic group on the surface of the nanoparticles. The band at 1434 cm^-1^ is associated with the asymmetric stretching vibration of the carboxylic group (-COO^-^) chelated with lanthanide ions, which causes the red-shift of the carboxylic group. The single -CO stretching band of malonic acid are situated at 1125 cm^-1^. Photoluminescence showed the upconversion of the NIR to green and red emission. Green emission at 540 nm is relatively stronger than red emission. Fe-SEM pictures confirms the cubic phase of UCNPs. Moreover, the size distribution of the UCNPs is 72 nm.

Synthesis of phosphate surfactant

Step 1: Synthesis and purification of Ethylene glycol stearate

In figure S2b, ^1^H NMR of ethylene glycol stearate illustrates a broad singlet peak between 1.26 and 1.31 ppm that corresponds to the hydrogens from a fatty acid chain and a triplet peak at 0.88 ppm that is associated with the methyl group of stearic acid. The multiplet present at 1.64 ppm is associated with the hydrogens on the β carbon whereas the hydrogens on the α carbon appear as a triplet at 2.32 ppm. Triplets at 3.65 and 4.20 ppm correspond to the methylene hydrogens of ethylene glycol.

As mentioned above, dicyclohexylurea (DCU) was still present in the product after washing with sodium bicarbonate and a dilute solution of hydrochloric acid, which can be seen in figure S3 with a peak appearing at 3326 cm^-1^ corresponding to the N-H stretch of DCU overlapping with the O-H stretch of EGS. Therefore, the obtained product in the form of crystals was washed with methanol, in which EGS was not soluble because of the presence of the long hydrocarbon chain. Since DCU is highly soluble in methanol, the product was washed with methanol for varying amounts of time. According to figure S3, 5 minutes of stirring in methanol was determined to be optimal because DCU was not removed completely if the product was washed for less than 5 minutes, but the product was damaged when washed longer than 5 minutes.

Step 2: Synthesis and purification of 2-(phosphonooxy)ethyl stearate

In figure S2b, proton NMR illustrates the formation of the phosphate surfactant. The triplet peak at 0.75 ppm is associated with the methyl group of stearic acid while a broad singlet peak between 1.12 and 1.16 ppm shows the fatty acid hydrogens. Peaks have been shifted to lower ppm due to the use of different solvents.

To confirm the presence of the phosphate functional group on the surfactant after monophosphorylation, elemental analysis (EDAX) of the surfactant was performed. In figure S4, EDAX results confirm the presence of carbon (C), Oxygen (O), and Phosphorus (P). Moreover, the percentages of atoms correlate with the structure of the surfactant.

Step 3: PEGylation of the surfactant

The PEGylated 2-(phosphonooxy)ethyl stearate surfactant was then characterized by FTIR, shown in figure S2. The presence of the peak at 1737 cm^-1^ corresponds to the ester group of the phosphate surfactant, and the strong peak situated between 3000 cm^-1^ and 2820 cm^-1^ corresponds to the increased number of aliphatic hydrocarbons. Moreover, peaks located between 1000 cm^-1^ and 1100 cm^-1^ confirm the presence of the P-O-C group, confirming the successful addition of polyethylene glycol to the phosphate group.

Activity of venom bee sPLA-2 on surfactant

The activity of enzyme sPLA-2 on the phosphate surfactant was confirmed through digestion and liquid chromatography-tandem mass spectrometry (LC/MS). However, after addition of the enzyme to the phosphate surfactant, we observe physical changes in the solution. Shown in figure S5b, after the addition of the sPLA-2 enzyme to a solution containing phosphate surfactant, we observed a formation of stearic acid suspended in water that migrated to the top of the tube. We suppose that after the digestion of phosphate surfactant, stearic acid, which is insoluble in water and has a relatively lower density compared to water, migrates to the top of the tube. Moreover, during the extraction step of the samples in dichloromethane (DCM) before injection in LC/MS, we observed that the aqueous phase of the treated sample was more transparent (Figure S5b), since the stearic acid suspended in water is highly soluble in DCM at low concentrations.

Encapsulation of UCNPs in micelles

Since the phosphate surfactant is soluble in water due to the PEG chains, UCNPs encapsulated with the surfactant dispersed very quickly compared to the free UCNPs. To confirm that UCNPS were successfully loaded in the micelles, FTIR was performed, as shown in Figure S6. The FTIR spectrum confirms the presence of the functional groups of the phosphate surfactant as well as UCNPs. The broad absorption band at around 3435 cm^-1^ corresponds to the O-H stretching vibration from UCNPs, and the peak at 1737 cm^-1^ is associated with the ester group of the phosphate surfactant. Moreover, to confirm the encapsulation of UCNPs, elemental analysis (EDAX) was performed. In Figure S7 the presence of all atoms from the UCNPs and phosphate surfactant illustrates that the encapsulation was successful. However, the peak intensity for the phosphorous atom is relatively low because the percentage of phosphorous is small compared to PEG and the long hydrocarbon chains of the surfactant. Copper ions also appeared on this EDAX spectra because the surfactant was deposed on copper tape.

Moreover, the dispersion of the MA@UCNPs in different solvents with polarity ranging from 0.1-10.2 was checked, see the Table S1. 3mg of UCNPs was dispersed in 2 ml of solvent and sonicated for 30 minutes. To compare the effect of the surfactant on the dispersion of UCNPs in highly polar solvents, methanol and water with 6 x 10^-4^ M surfactant were prepared, and the UCNPs were added at same time in all solvents. The results, showed that the dispersion of UCNPs in n-Hexane, carbon tetrachloride and chloroform is complete and very fast. However, the dispersion of the UCNPs in methanol and water is respectively partial and low, even after a long-time sonication. Moreover, methanol and water solvents containing surfactant showed a complete and fast dispersion of UCNPs as shown in the Figure S8. Additionally, the mechanism of the liberation of UCNPs in presence of sPLA-2 suggests the formation of micelles.

In vitro bioimaging application

The bioimaging was performed on three different cells: HeLa (Human cervical cancer, adenocarcinoma), KB (HeLa contaminant, carcinoma) and 22Rv1 (prostate carcinoma). UCNPs encapsulated in micelles showed a selectivity toward the 22Rv1 cell line. However, these UCNPs without encapsulation show affinity to all cells. Figure S9 shows the in vitro bioimaging of the HeLa and KB cell lines performed with UCNPs without encapsulation in micelles. As we can see, both cells were wrapped with UCNPs and have a high upconversion fluorescence imaging. We can confirm that without a delivery system UCNPs have no selectivity and can bind to any cells.

Cytotoxicity of UCNPs compared with UCNPs-loaded micelles

Cytotoxicity is a critical assessment for all new emerging nanomaterials, especially those which are applied in nanomedicine. Herein, MTT assay results showed a reduction of the toxicity of surfactant-encapsulated UCNPs towards three different cells when compared to free UCNPs. When using a concentration of 0.300 mg/mL of UCNPs and 2.27 μM of phosphate surfactant rather than free UCNPs, the cell viability increased from 86.23 % to 94.21 %, from 76.49 % to 89.65 %, and from 71.60 % to 78.07 % for HeLa, KB, and 22Rv1 cell lines, respectively (Figure S10). When a lower concentration of 0.075 mg/mL of UCNPs and 0.5675 μM of phosphate surfactant was tested, the cell viability increased from 93.19 % to 97.98 %, from 85.39 % to 96.81 %, and from 83.86 % to 84.98 % for HeLa, KB, and 22Rv1 cell lines, respectively. As seen in Figure S10, the 22Rv1 cell lines showed a lesser increase in cell viability than HeLa and KB cell lines when treated with surfactant-encapsulated UCNPs, which can be explained by the cleavage of the phosphate surfactant by the overexpressed sPLA-2 enzyme and further liberation of UCNPs in high concentrations on the surface of the cells, further indicating the specificity of the surfactant-encapsulated UCNPs towards the 22Rv1 cell line.


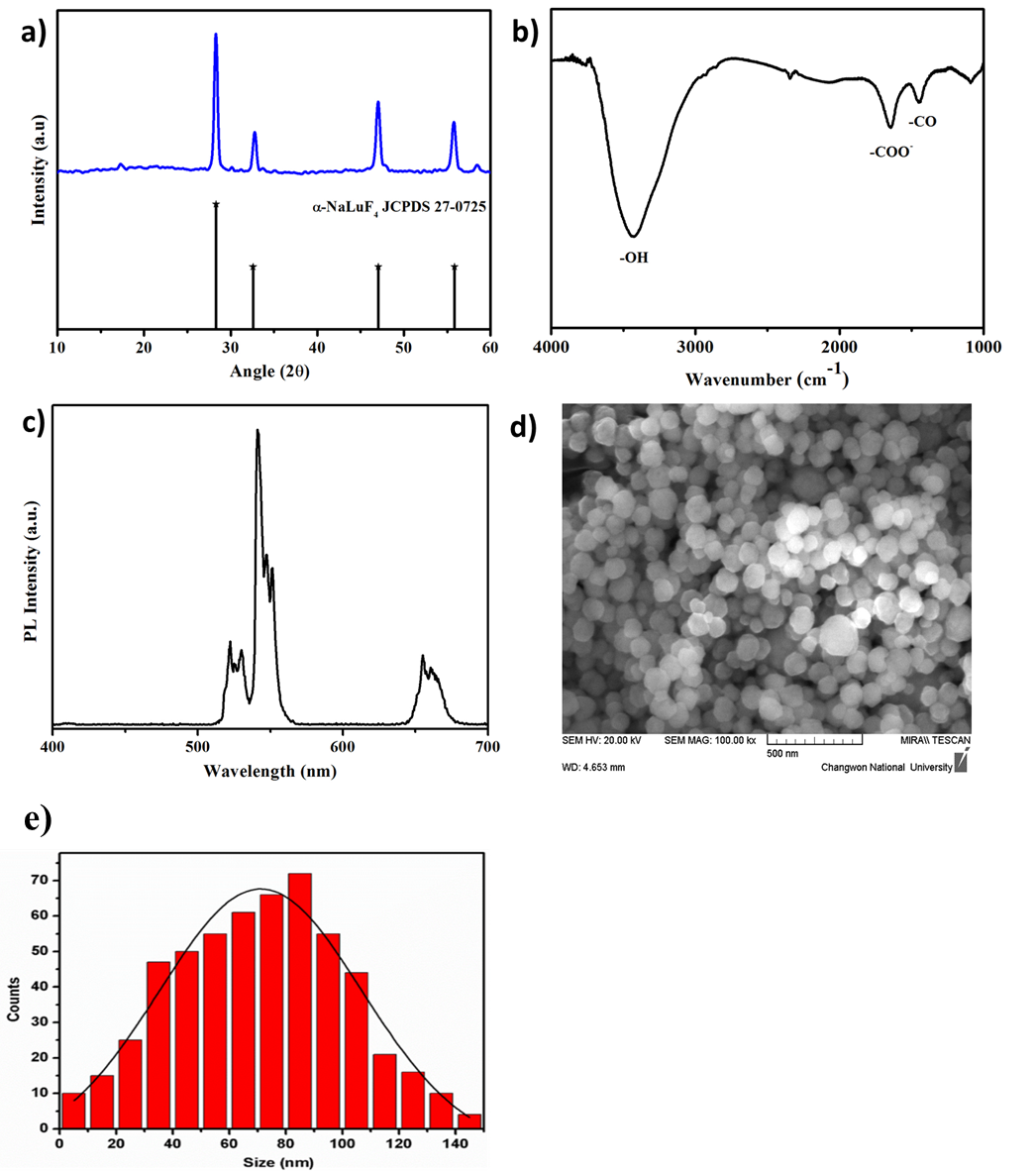
Figure S1. Characterization of Carboxyl-functionalized NaLuF4:Gd^3+^/Yb^3+^/Er^3+^ upconversion nanoparticles. a) XRD pattern of UCNPs. b) FT-IR spectrum of UCNPs. c) Photoluminescence spectra of UCNPs. d) Fe-SEM image of UCNPs. e) Size distribution of UCNPs. The average size of UCNPs was found to be 72 nm.

Figure S2. Characterization of the phosphate surfactant. a) FTIR spectra of synthesized surfactant in three steps. First (top) is EGS after esterification, second (middle) is 2-(phosphonooxy)ethyl stearate after phosphorylation of EGS, and third (bottom) represent final phosphate surfactant after PEGylation. b) ^1^H NMR of the surfac
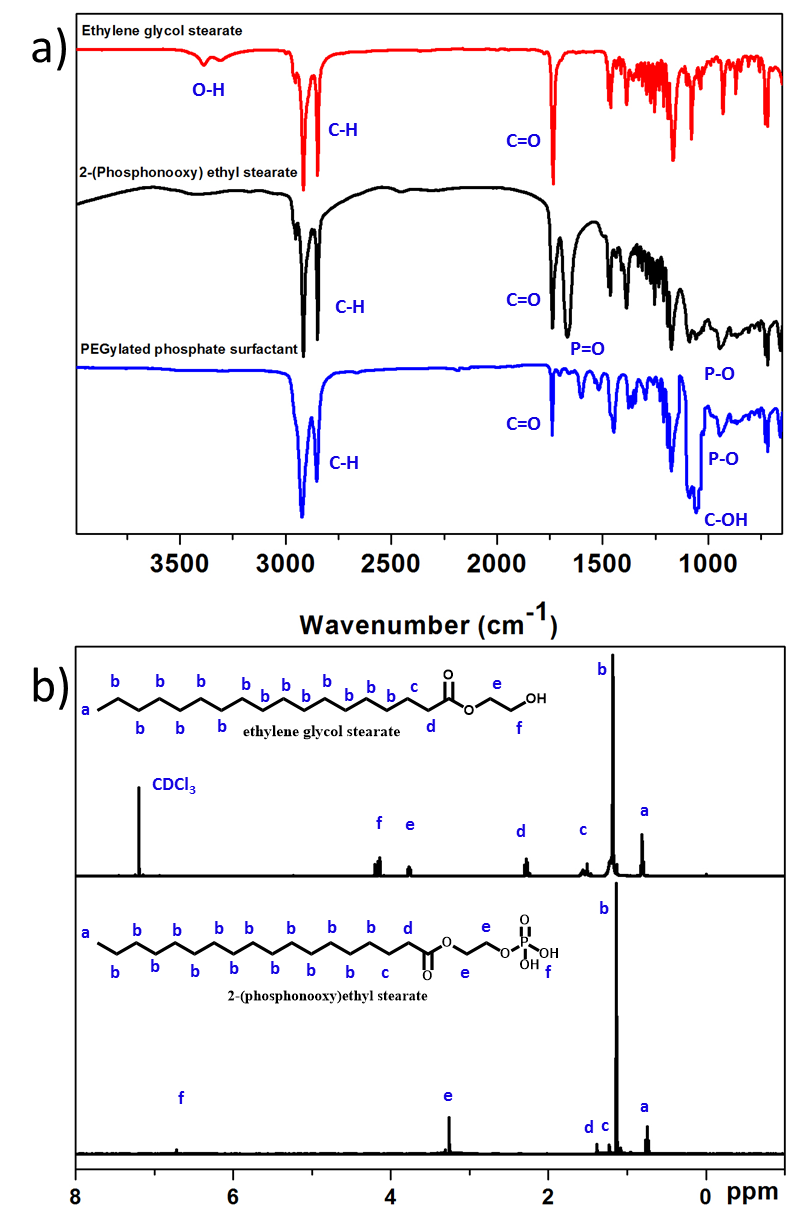
tant for first and second steps.

Figure S3. FT-IR spectra of ethylene glycol stearate after washing with methanol. EGS was washe
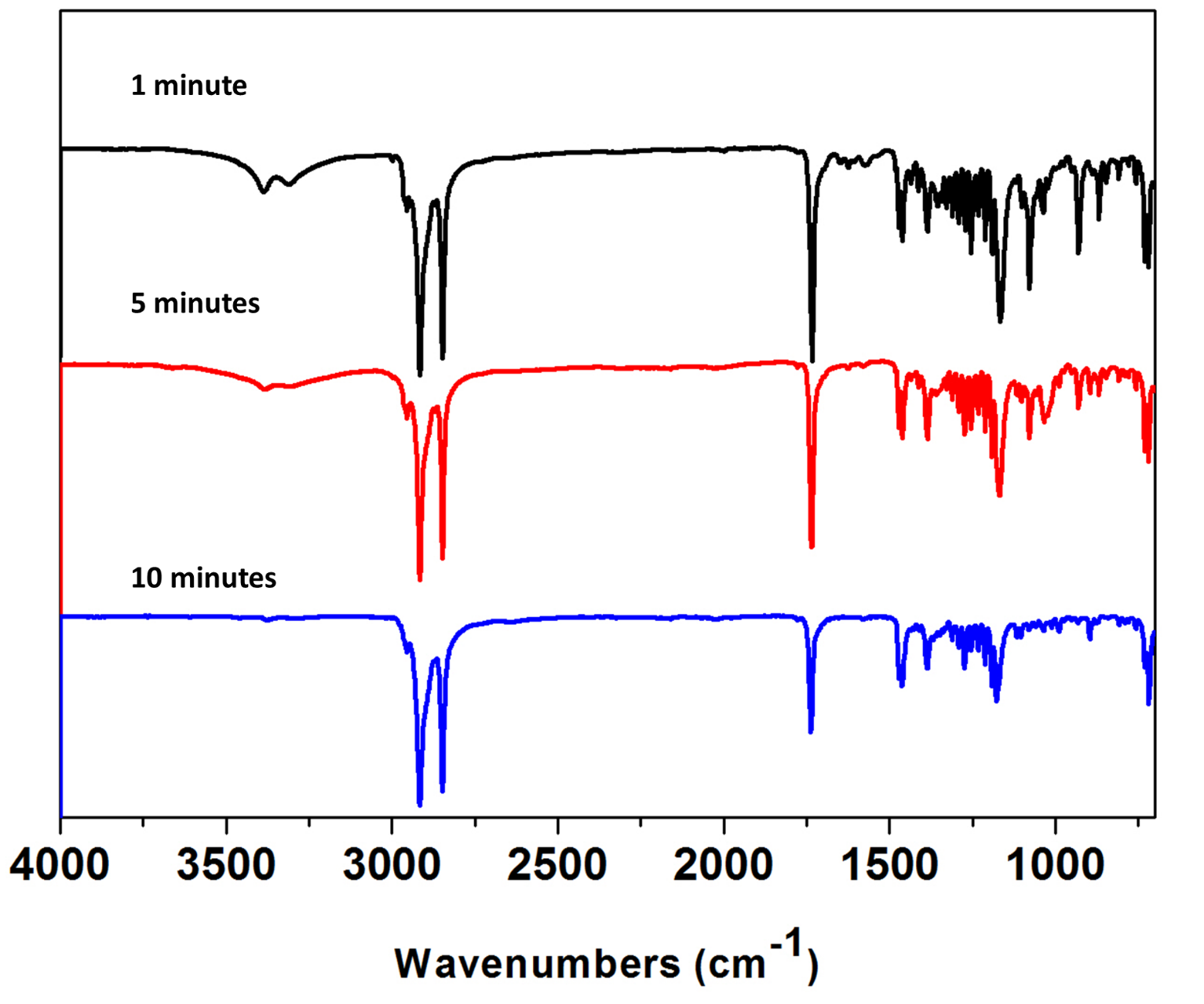
d with methanol for varying times to remove DCU. Optimized time of washing was found to be 5 mins.

Figure S4. Elemental analysis (EDAX) of surfactant after monophosphorylation. The presence of
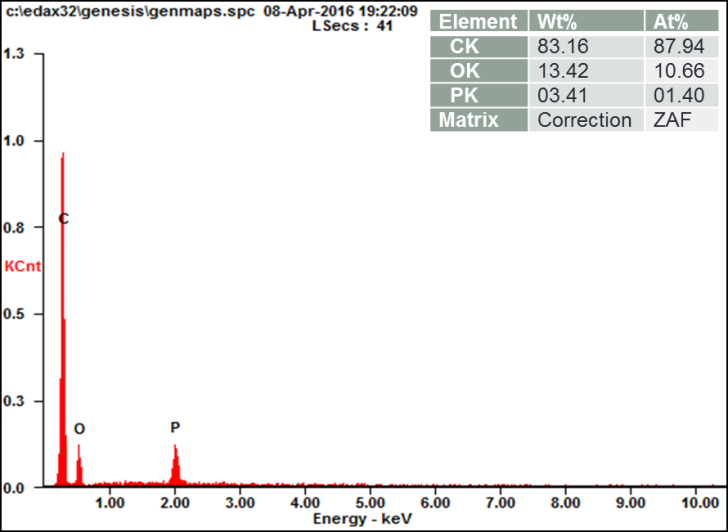
 carbon, oxygen and phosphorous atoms confirm the structure of 2-(phosphonooxy) ethyl stearate.

Figure S5. Picture of physical changes revealed after digestion. a) Surfactant dissolved in water with and without treatment by sPLA-2 (60-240
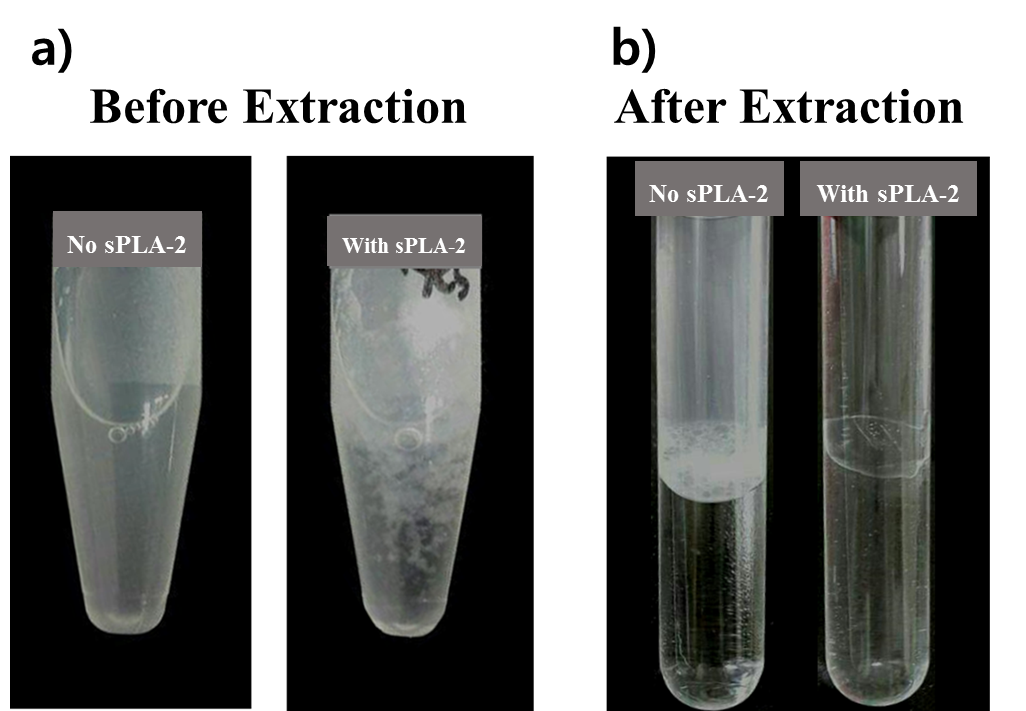
 U/L) before extraction with DCM . b) Surfactant dissolved in water with and without treatment by sPLA-2 (60-240 U/L) after extraction with DCM


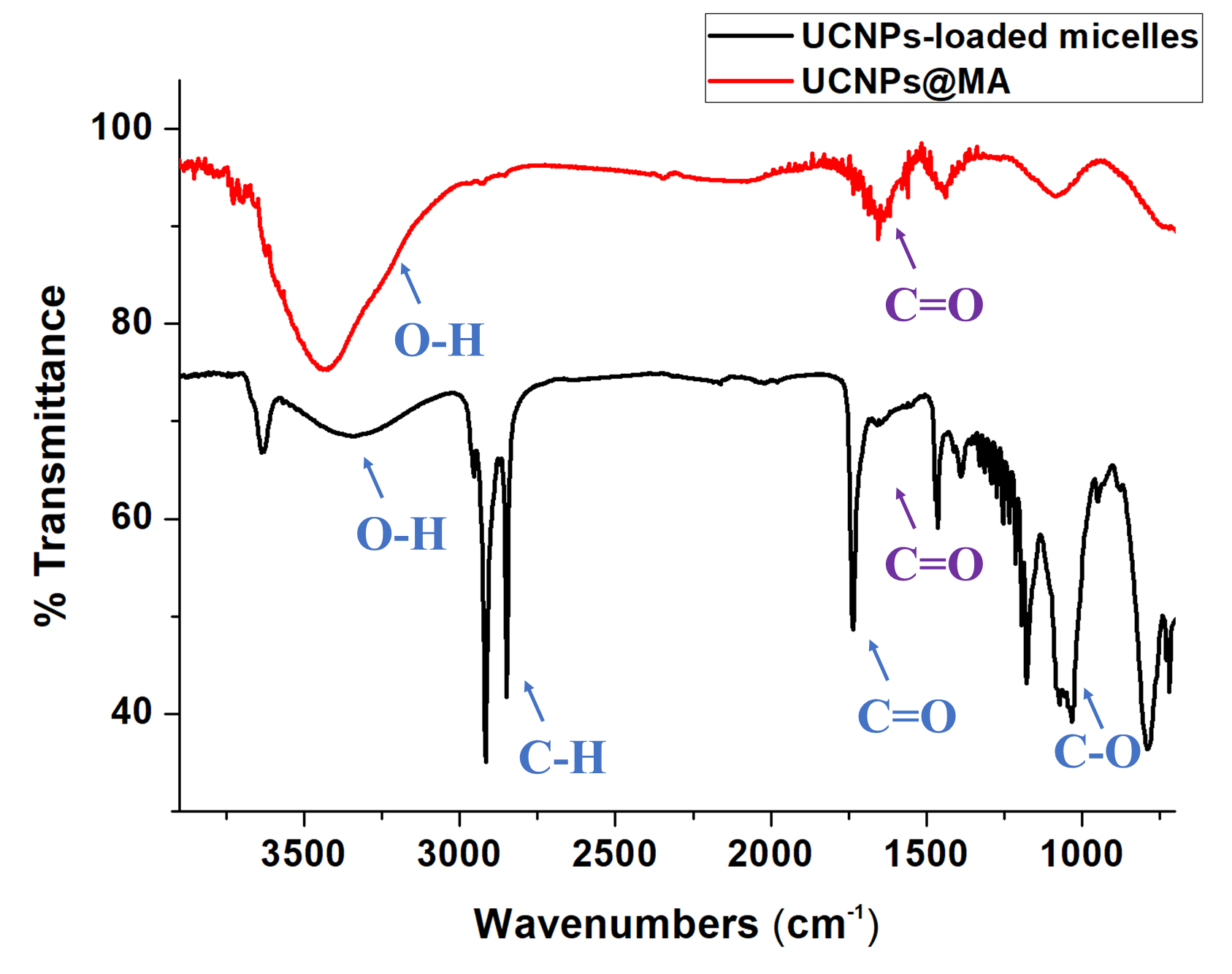


**Figure S6. FTIR spectrum of UCNP@MA and UCNP@MA-loaded micelles.** (Red) FTIR spectrum of UCNP@MA. The peak at 1652 cm^-1^ (purple) corresponds to carboxylic function of the malonic acid. (Black) FTIR spectrum of the UCNP@MA-loaded micelles. The peak at 1652 cm^-1^ exclude the formation of H-bonds between UCNP@MA and Surfactant.

Figure S7. Elemental analysis of NaLuF4:Gd^3+^/Yb^3+^/Er^3+^ UCNP-loaded micelles.


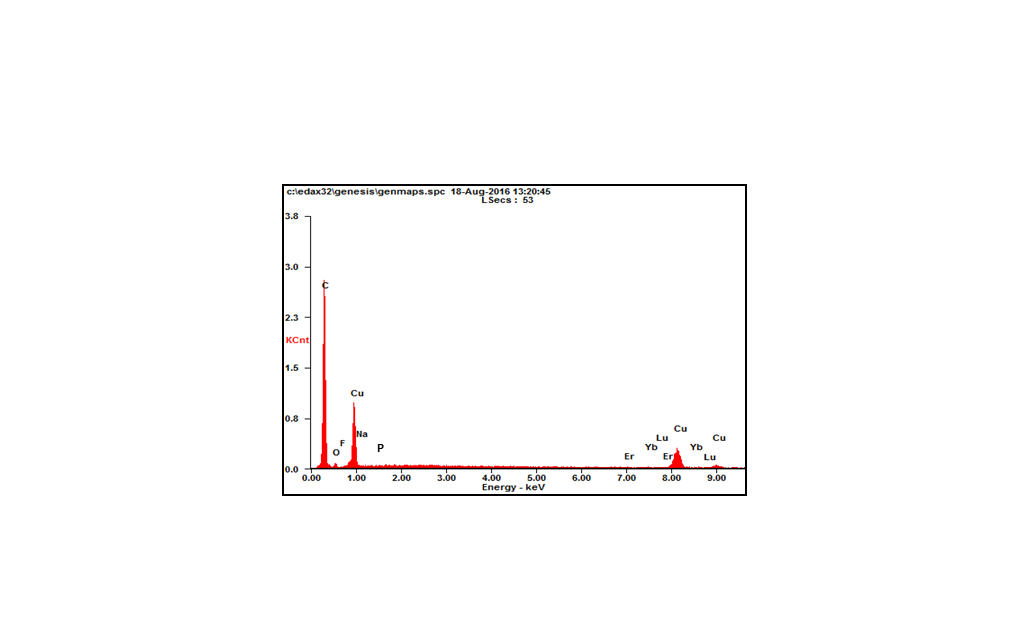


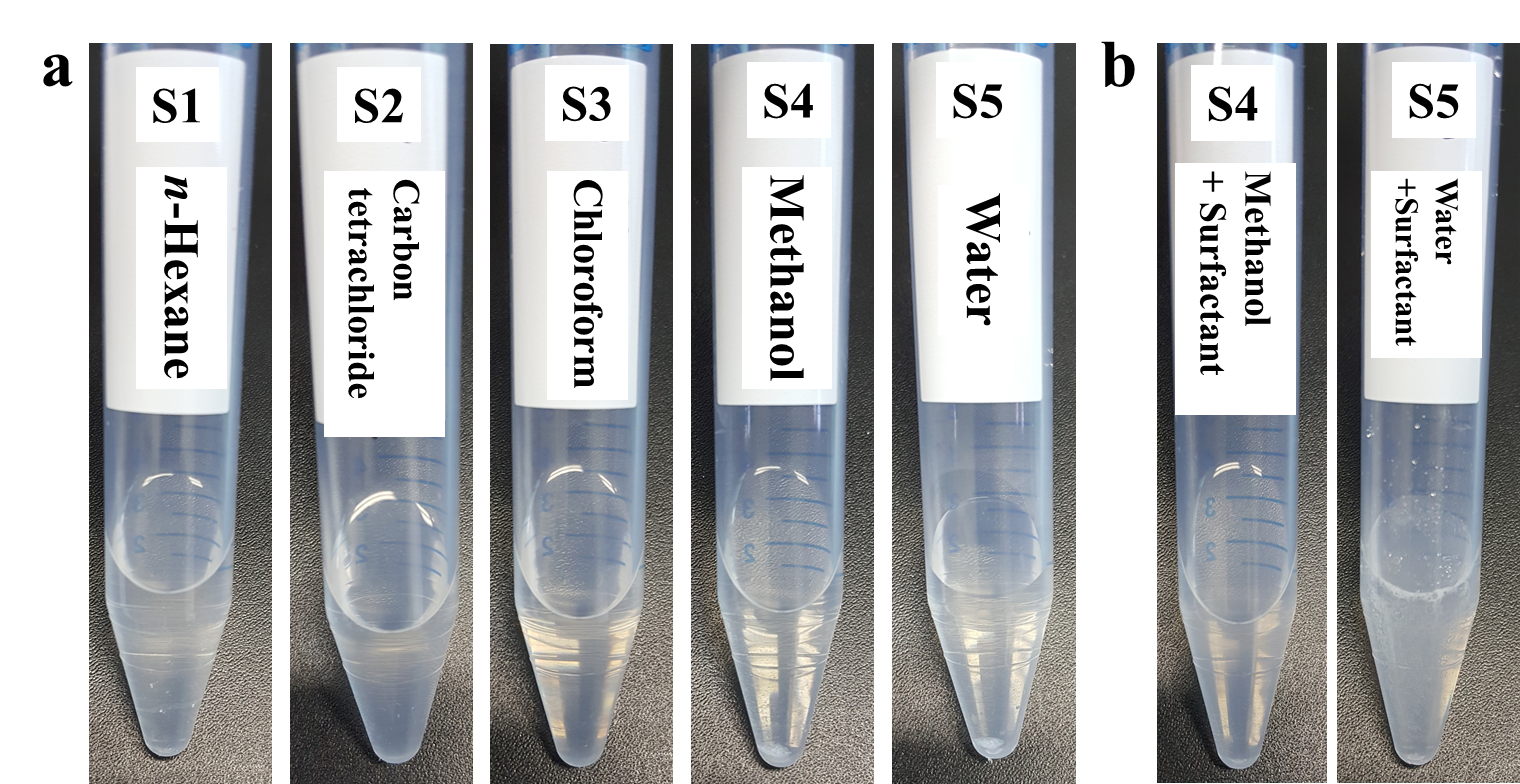
Figure S8. **Dispersion of UCNPs and UCNPs-loaded micelles in different solvents**. (a) Dispersion of UCNPs in different solvents with polarity ranging from 0.1 to 10.2 (b) The Complete dispersion of UCNPs in methanol and water in the presence of the surfactant.

**Table S1. Dispersion of UCNPs and UCNPs-loaded micelles in different solvents with polarity ranging from 0.1 to** 10.2

| **Solvents** | **Polarity** | **UCNPs** | **UCNPs with Surfactant** |
| --- | --- | --- | --- |
| *n*-Hexane | 0.1 | Dispersed |  |
| Carbon tetrachloride | 1.6 | Dispersed |  |
| Chloroform | 4.1 | Dispersed |  |
| Methanol | 5.1 | Partially dispersed | Dispersed |
| Water | 10.2 | Low dispersion | Dispersed |

Figure S9. Bioimaging of Hela and KB Cells with UCNPs without further encapsulation in surfactant micelles a) HeLa cells treated with UCNPs b) KB cells treated with UCNPs.


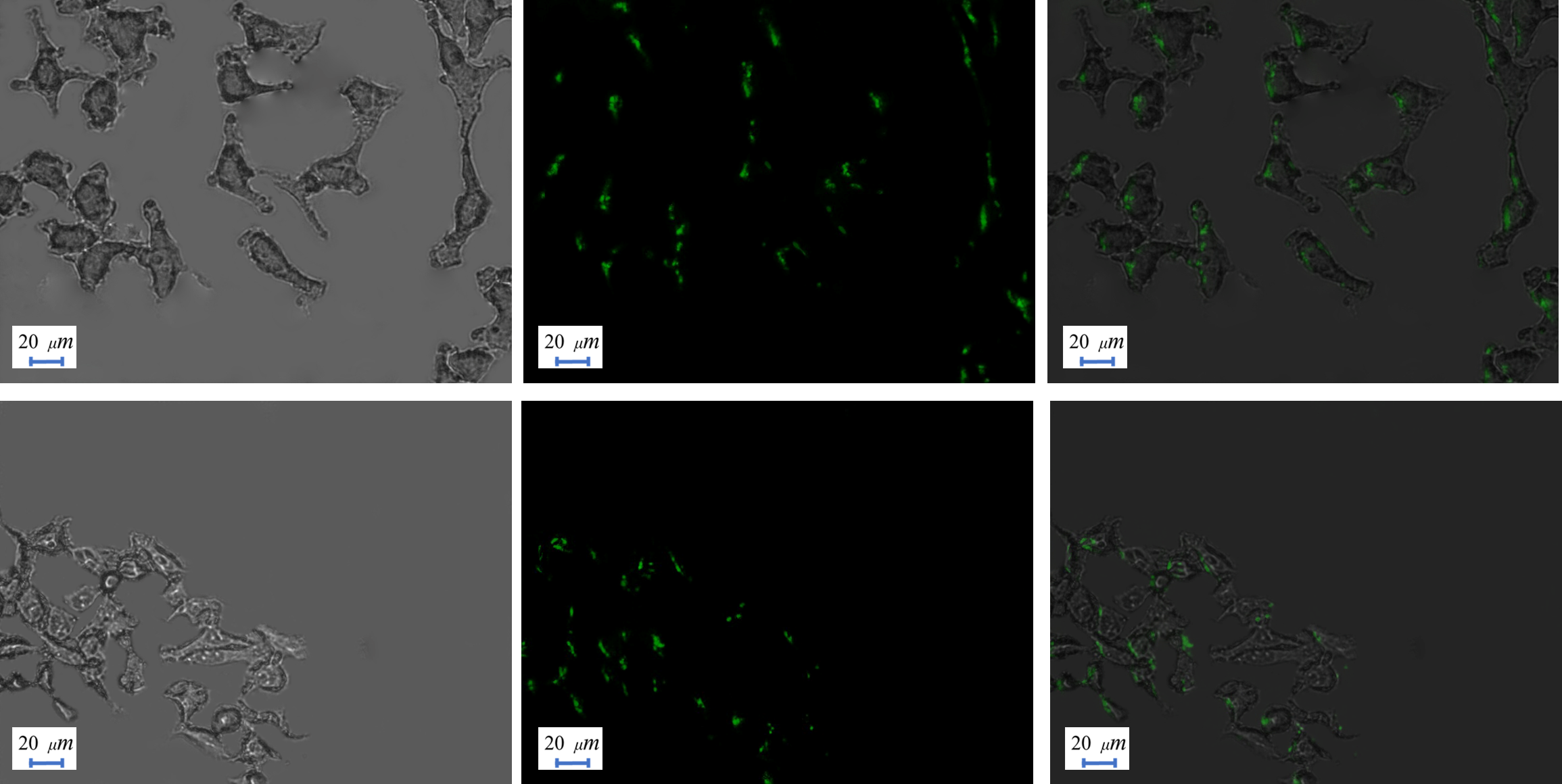


Table S2. Comparison of average cell viability for three different cell lines in presence of 300μg/mL UCNPs and UCNP-loaded micelles. Encapsulated UCNPs showed a lower cytotoxicity compared to free UCNPs.

|  | HeLa^[a]^ | KB^[b]^ | 22Rv1^[c]^ |
| --- | --- | --- | --- |
| UCNPs | 86.23% | 76.49% | 71.60% |
| UCNPs-loaded micelles | 94.21% | 89.65 | 78.07% |

[a] HeLa cell line incubated with 300μg/mL of UCNPs with and without encapsulation. [b] KB cell line incubated with 300μg/mL of UCNPs with and without encapsulation. [c] 22Rv1 cell line incubated with 300μg/mL of UCNPs with and without encapsulation.

Figure S10. MTT assay of UCNPs with and without further encapsulation on three various cells. Human cervical cancer (HeLa), HeLa contaminant (KB) and Human prostate cancer (22R
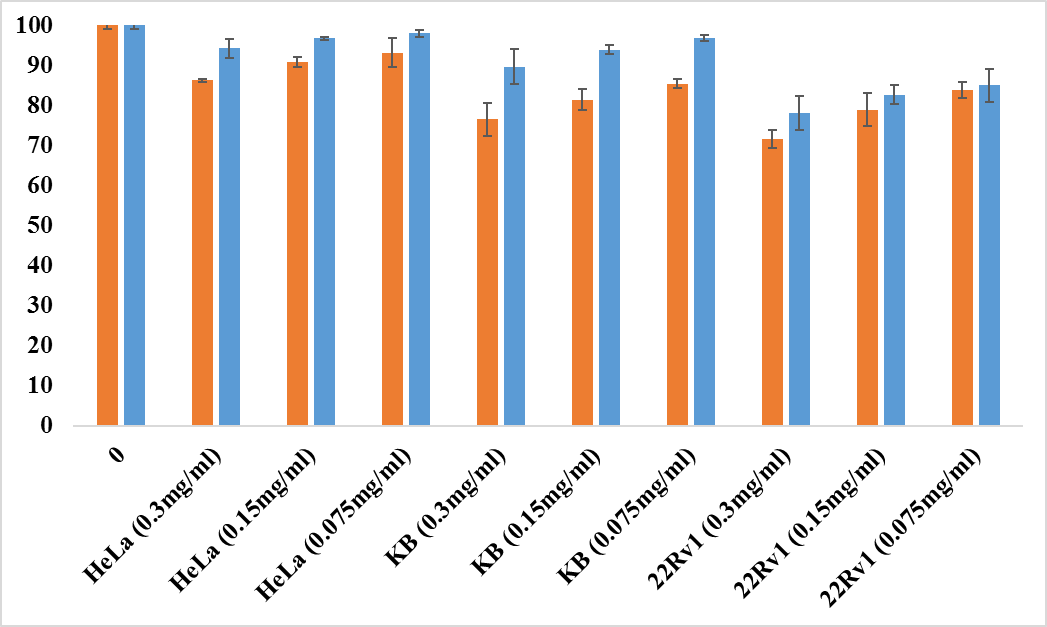
v1) cell lines were treated with UCNPs (orange) and UCNP-loaded micelles (blue). Average cell viability showed a reduction of cytotoxicity of UCNPs after loading in micelles.

References

1. Yang, Y. *et al.* Hydrothermal synthesis of NaLuF4:153Sm,Yb,Tm nanoparticles and their application in dual-modality upconversion luminescence and SPECT bioimaging. *Biomaterials* **34,** 774–783 (2013).

2. Xiong, L.-Q. *et al.* Synthesis, characterization, and in vivo targeted imaging of amine-functionalized rare-earth up-converting nanophosphors. *Biomaterials* **30,** 5592–5600 (2009).

3. Neises, B. & Steglich, W. Simple Method for the Esterification of Carboxylic Acids. *Angew. Chemie Int. Ed. English* **17,** 522–524 (1978).

4. Sakakura, A., Katsukawa, M. & Ishihara, K. Selective synthesis of phosphate monoesters by dehydrative condensation of phosphoric acid and alcohols promoted by nucleophilic bases. *Org. Lett.* **7,** 1999–2002 (2005).
